# ROS-mediated activation of *Drosophila* larval nociceptor neurons by UVC irradiation

**DOI:** 10.1186/1471-2202-15-14

**Published:** 2014-01-16

**Authors:** Myung-Jun Kim, Wayne A Johnson

**Affiliations:** 1 Department of Molecular Physiology and Biophysics, University of Iowa, Roy J. and Lucille A. Carver College of Medicine, Iowa City, IA 52242, USA; 2 Current address: Department of Genetics, Cell Biology and Development, University of Minnesota, Minneapolis, MN, USA

**Keywords:** Sensory signaling, Reactive oxygen species, *Drosophila*, Behavior

## Abstract

**Background:**

The complex *Drosophila* larval peripheral nervous system, capable of monitoring sensory input from the external environment, includes a family of multiple dendritic (md) neurons with extensive dendritic arbors tiling the inner surface of the larval body wall. The class IV multiple dendritic (mdIV) neurons are the most complex with dendritic nerve endings forming direct intimate contacts with epithelial cells of the larval body wall. Functioning as polymodal mechanonociceptors with the ability to respond to both noxious mechanical stimulation and noxious heat, the mdIV neurons are also activated by nanomolar levels of the endogenous reactive oxygen species (ROS), H_2_O_2_. Although often associated with tissue damage related to oxidative stress, endogenous ROS have also been shown to function as signaling molecules at lower concentrations. The overall role of ROS in sensory signaling is poorly understood but the acutely sensitive response of mdIV neurons to ROS-mediated activation is consistent with a routine role in the regulation of mdIV neuronal activity. Larvae respond to short wavelength ultraviolet (UVC) light with an immediate and visual system-independent writhing and twisting of the body previously described as a nociceptive response. Molecular and cellular mechanisms mediating this response and potential relationships with ROS generation are not well understood. We have used the UVC-induced writhing response as a model for investigation of the proposed link between endogenous ROS production and mdIV neuron function in the larval body wall.

**Results:**

Transgenic inactivation of mdIV neurons caused a strong suppression of UVC-induced writhing behavior consistent with a key role for the mdIV neurons as mediators of the behavioral response. Direct imaging of ROS-activated fluorescence showed that UVC irradiation caused a significant increase in endogenous ROS levels in the larval body wall and transgenic overexpression of antioxidant enzymes strongly suppressed the UVC-induced writhing response. Direct electrophysiological recordings demonstrated that UVC irradiation also increased neuronal activity of the mdIV neurons.

**Conclusions:**

Results obtained using UVC irradiation to induce ROS generation provide evidence that UVC-induced writhing behavior is mediated by endogenous production of ROS capable of activating mdIV mechanonociceptors in the larval body wall.

## Background

Longterm survival of organisms living in constant contact with a highly stimulating external environment depends upon the efficient distinction between beneficial and hazardous signals. Food-associated cues, for example, are likely to indicate favorable conditions while stimuli capable of causing painful sensations and/or tissue damage should prompt an aversive avoidance behavior. Consequently, animals are equipped with specific sensory neurons called nociceptors to detect noxious stimuli and elicit protective behavioral responses using combinations of thermal, mechanical and/or chemical signals [1].

The body wall of *Drosophila* larvae is comparable to vertebrate skin containing a variety of sensory neurons and associated structures [2-4]. The class IV multiple dendritic (mdIV) neurons extend complex dendritic arbors to completely tile the inner surface of the body wall [3,5]. These neurons express the *Drosophila* Degenerin/Epithelial Sodium Channel (DEG/ENaC) subunit Pickpocket1 (PPK1) [6-8] and function as nociceptors in the body wall where they mediate thermal and mechanical nociceptive behaviors [9,10]. PPK1 is necessary for the mechanical nociception response but is dispensable for thermal nociception [9,10].

We have previously characterized an mdIV neuron-dependent hyperoxia aversion behavior in foraging stage larvae and demonstrated that it is mediated by detection of the reactive oxygen species (ROS), H_2_O_2_[11]. Previous studies have also shown that *Drosophila* wandering stage larvae exhibit immediate writhing motion upon exposure to short wavelength ultraviolet radiation (UVC) [12]. Transgenic disruption of the visual system did not lead to suppression of the writhing behavior and restricted irradiation at any position in the body wall where nociceptors extend extensive dendritic arbors could elicit writhing. Based on these observations, the UVC-induced writhing motion has been classified as nociception behavior. Recently, illumination with light of longer wavelength (from blue light to UVA) has been shown to cause light-avoidance behavior without apparent induction of writhing motion in *Drosophila* larvae [13]. Thus, ultraviolet radiation (UVR) appears to elicit distinct behavioral responses from *Drosophila* larvae depending on the wavelength.

UVR is an important environmental threat that causes both acute and chronic skin problems such as sunburn, pigmentation, immunosuppression, sensitization to upcoming stimuli, photoaging and cancer in various animal species [14-17]. UVR is divided into three major categories based upon wavelength [18]. UVC has a short wavelength (190–280 nM) and is completely absorbed by molecular oxygen in the atmosphere so that it usually does not reach the earth’s surface [19]. UVB has an intermediate wavelength of 280–320 nm and, although it is largely absorbed by the ozone layer, some portion is known to reach the ground [18]. UVA, classified as 320–400 nM, easily penetrates the atmosphere and is a major form of UVR in sunlight [20]. Each class of UVR exerts adverse effects on skin through distinct but somewhat overlapping molecular mechanisms. For example, UVA and UVB mainly promote ROS production leading to oxidative damage of macromolecules and cell apoptosis [21-25]. In addition, UVB can cause DNA lesions by inducing the formation of cyclobutane-pyrimidine dimers and pyrimidine-pyrimidone photoproducts [26,27]. Like UVB, UVC causes tissue responses and DNA damage similar to UVB, but its effects are more severe [18].

Mammalian experimental models have long been a focus in the study of UVR-related disorders. Recently, however, *Drosophila* has proven useful as a genetic model system for this type of analysis with the appreciation that the molecular and cellular mechanisms mediating the UVR response are largely conserved between insects and mammals in spite of their differences in integument structures. For example, like its mammalian counterpart, the *Drosophila* p53 protein plays a pivotal role in the response to DNA damage caused by UVR [28,29]. *Drosophila* larvae have also been shown to display nociceptor sensitization after UVR exposure resulting in allodynia and hyperalgesia similar to that observed in vertebrates [30]. This UVR-induced sensitization relies on intercellular communication between epidermal cells and peripheral neurons using *Drosophila* tumor necrosis factor (TNF) as a signaling molecule. TNF-mediated intercellular communication has been shown to play a major role in the development of inflammation and hyperalgesia following UVR in mammalian integuments [31,32]. Taken together, these observations suggest that UVR activates similar cellular and molecular events in vertebrate and invertebrate integuments. The availability of powerful genetic tools in the *Drosophila* system and the sharing of molecular and cellular mechanisms between Drosophila and mammalian UVR responses raise the exciting possibility that *Drosophila* can serve as an excellent model for studying the effect of UVR on animals.

The strong larval writhing behavioral response to UVR is similar to behaviors elicited by noxious heat mediated by the mdIV sensory neurons [10,33]. Previous work has linked UV irradiation to the production of ROS [21-25] and our published data demonstrated a hyperoxia aversion behavior mediated by ROS-dependent activation of mdIV sensory neurons [11]. In light of these results, we have examined whether the observed UVR-induced larval writhing behavior is also mediated by an ROS-dependent activation of the mdIV sensory neurons in the larval body wall.

Here we demonstrate a crucial role for mdIV sensory neurons in the expression of writhing motion upon UVC irradiation in *Drosophila* larvae. We also show that ROS production is required to induce the writhing motion. Results from direct electrophysiological recordings showed that UVC increases the neuronal activity of mdIV neurons. These results suggest that UVC irradiation promotes ROS generation in the larval integument system leading to the activation of mdIV neurons and subsequent onset of the writhing motion.

## Results

### UVC induces mdIV-mediated writhing behavior

**Figure 1:**
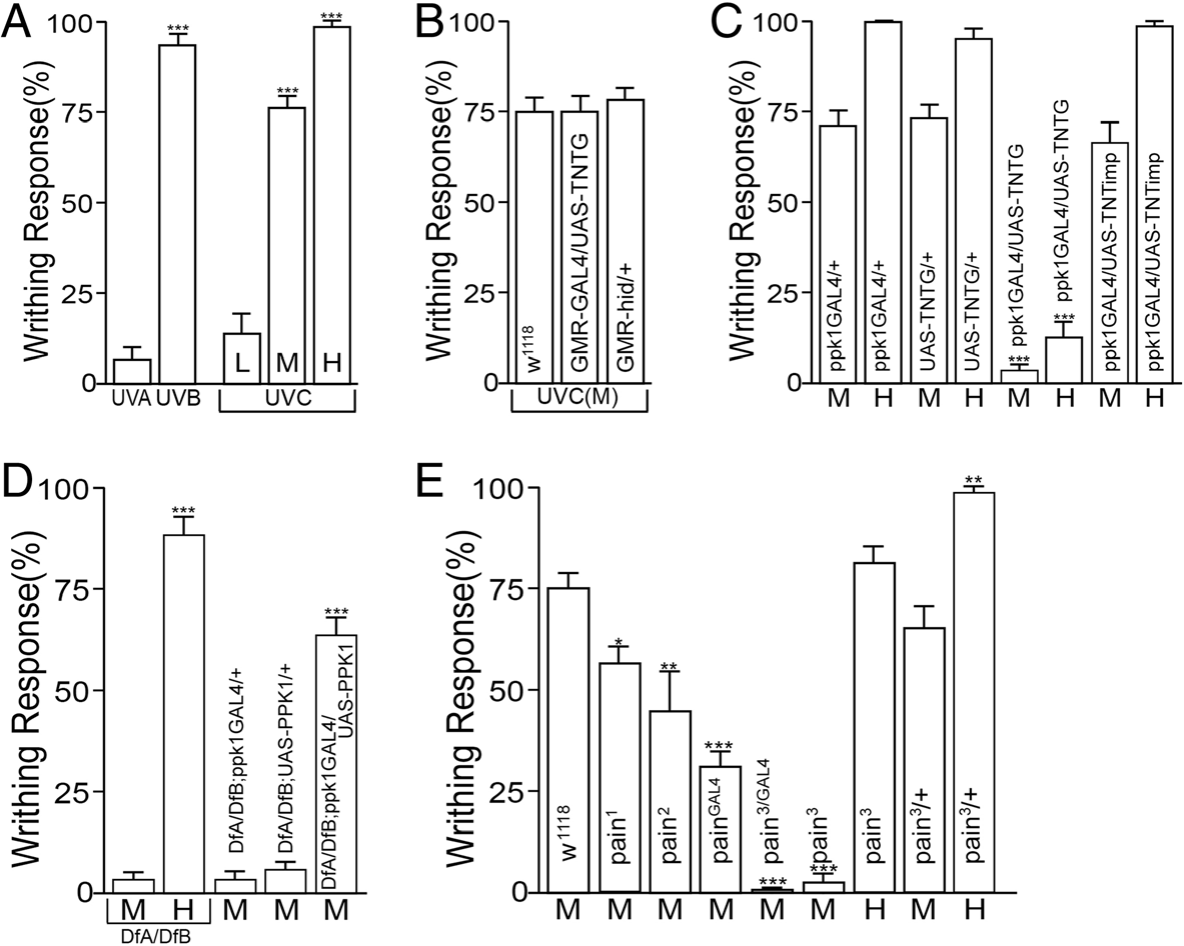
**Induction of mdIV neuron-dependent larval writhing behavior by UV irradiation. (A)** Larval writhing behavior in response to UV irradiation. High intensity UVB and UVC exposure induced robust writhing behavior w^1118^ 3rd instar larvae. Exposure to UVA did not cause a significant behavioral response. Varied intensities of UVC irradiation resulted in a dose-dependent response(n = 6, 9, 8, 15 and 10, respectively). ***p < 0.0001, one-way ANOVA with Tukey’s post comparison test vs. UVA **(B)** Transgenic inactivation of the larval visual system in GMR-GAL4/UAS-TNT-G and GMR-hid larvae did not suppress the writhing behavior after exposure to UVC(M)(n = 6 for all). **(C)** Inactivation of larval mdIV neurons in ppk1GAL4/UAS-TNT-G larvae resulted in a greatly reduced response to both UVC(M) and UVC(H)(n = 6, 6, 5, 9, 9, 11, 6 and 8, respectively). ***p < 0.0001, one-way ANOVA with Tukey’s post comparison test vs ppk1GAL4/+(H/M). **(D)***ppk1* null mutant(DfA/DfB) larvae showed a strongly reduced response to UVC(M) but responded normally to UVC(H). mdIV neuron-specific expression of PPK1 rescued the larval response to UVC(M)(n = 7, 9, 6, 7 and 6, respectively). ***p < 0.0001, one-way ANOVA with Tukey’s post comparison test vs DfA/DfB(M) **(E)***painless* mutant alleles displayed a reduced sensitivity to UVC(M) with a range of severity consistent with the phenotypic strength of the allelic series. The most severe allele, pain^3^, responded normally to UVC(H)(n = 14, 15, 6, 8, 9, 8, 27, 10 and 12, respectively). *p < 0.01, **p < 0.0001 ***p < 0.0001, one-way ANOVA with Tukey’s post comparison test vs w^1118^(M). All data are presented as mean±SEM. Each N value represents results from one experimental trial using 5 larvae exposed to UVR and visually scoring the percentage responding.

UVC has been shown to induce an immediate behavioral response from *Drosophila* larvae consisting of repeated vigorous bending of the body from side to side and referred to as writhing behavior [12]. This response is essentially identical to the previously characterized writhing response to noxious heat stimulus [10,33] (Additional files 1 and 2). To quantify the response, 5 larvae at a time were exposed to UVC and those displaying writhing behavior within 5 seconds were counted. The dosage effect of UVC was investigated using three different intensities of UVC. Exposure to 0.17 mW/cm^2^, hereafter referred to as UVC (L), induced writhing behavior from 20% of the larvae (Figure 1A). The percentage of larvae showing writhing behavior increased with higher intensities of UVC demonstrating a clear dosage effect with ~75% displaying writhing motion in response to 1.2 mW/cm^2^ (UVC(M)) and essentially 100% responding to 8 mW/cm^2^ (UVC(H)) (Figure 1A, Additional file 1). UVA and UVB were then tested for their ability to induce the writhing behavior. UVA failed to induce the writhing response even at high intensity but UVB was as effective as UVC in eliciting the writhing behavior (Figure 1A). This is consistent with previous findings [13] demonstrating that illumination in the range of blue light to UVA causes light-avoidance behavior without eliciting writhing motion. These findings also highlight that UVB and UVC induce the same class of behavioral response and endorses the use of UVC as a representative of short wavelength UV. Finally, blockade of synaptic transmission with transgenic expression of the active form of tetanus toxin (TNTg) or ablation of visual system function in GMR-GAL4/UAS-TNT-G and GMR-hid larvae did not suppress the UVC-induced writhing motion (Figure 1B) demonstrating the visual system-independence of this response [12].

Based on the observations that a UVR response is independent of the visual system and writhing motion is not observed under normal conditions, we hypothesized that the writhing behavior is a pain response. Since the mdIV peripheral sensory neurons in the larval body wall are known to be major nociceptors for noxious thermal and mechanical stimuli [9,10], we assessed their possible role in mediating the UVR-induced writhing response. Neuron-specific inactivation of mdIV neurons in ppk1GAL4/UAS-TNT-G larvae eliminated the response to UVC(M) and caused a greatly diminished response to UVC(H) (Figure 1C, Additional file 1). Expression of inactive TNT (TNTimp) had no effect (Figure 1C). These results imply that the mdIV neurons play an essential role in eliciting writhing motion upon UVC irradiation.

Previous work has shown that the DEG/ENaC channel subunit, Pickpocket1 (PPK1), is expressed specifically in mdIV neurons and is required for mechanical nociception [10] but was dispensable for thermal nociceptive behavior [10]. Thus, the role of PPK1 in nociception appears to be context-dependent. These results prompted us to examine whether PPK1 is necessary or dispensable for UVC-induced writhing behavior. *ppk1* null mutant larvae showed almost no response to UVC(M) but responded robustly to UVC(H) (Figure 1D). These results suggest that PPK1 is involved in producing the UVR-induced writhing behavior. However, it does not appear to be requisite for the response since the inhibitory effect of its absence can be overcome by UVC(H). Finally, mdIV neuron-specific expression of PPK1 using the GAL4/UAS system was able to rescue the reduced response of *ppk1* null mutant larvae to UVC(M) (Figure 1D). Transgenic rescue of the phenotype confirms that removal of *ppk1* is responsible for the defective UVC response.

Painless, a *Drosophila* TRP channel expressed in mdIV neurons, has been shown to be crucial for both mechanical and thermal nociception behaviors [10,33]. The role of Painless in the UVR response was evaluated using four different alleles, pain^1^, pain^2^, pain^3^ and pain^GAL4^ and a heteroallelic combination of pain^3^ and pain^GAL4^. Of the mutant combinations tested, pain^3^ had the most profound effect suppressing the response to UVC(M) to 4% (Figure 1E). As observed in *ppk1* mutants, *pain*^*3*^ larvae exhibited robust writhing behaviors upon irradiation with UVC(H) (Figure 1E). Other *pain* mutant alleles tested showed varying levels of suppression consistent with an allelic series of phenotypes and demonstrating that the observed *pain*^*3*^ response was not allele-specific (Figure 1E). A heteroallelic combination of pain^3^ and pain^GAL4^ displayed the strongest response with complete suppression of the medium intensity UVC-induced writhing behavior (Figure 1E).

### Generation of ROS is necessary for the UVR response

**Figure 2:**
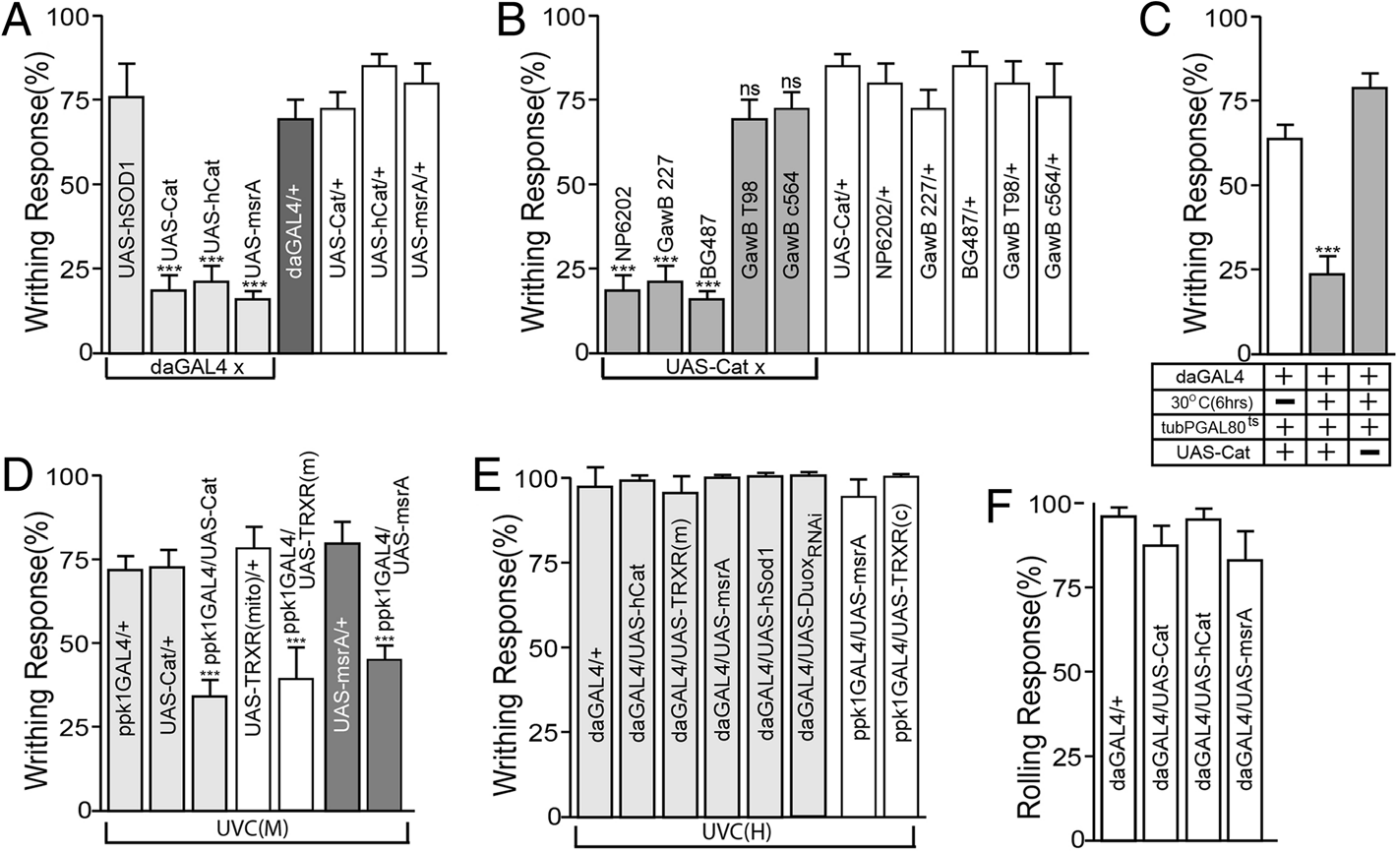
**Writhing behavior induced by UVC(M) requires generation of endogenous ROS. (A)** Ubiquitous expression of antioxidant enzymes in transgenic larvae suppresses the behavioral response induced by medium intensity UVC(M). Superoxide dismutase(hSod1) had no effect on the response(n = 5, 14, 11, 10, 8, 6, 6 and 6, respectively). ***p < 0.0001, one-way ANOVA with Tukey’s post comparison test, daGAL4/UAS vs UAS/+ . **(B)** Tissue-specific expression of catalase enzyme to degrade endogenous H_2_O_2_ efficiently suppressed UVC(M)-induced writhing behavior only when expressed in or near the epidermis (NP6202, GawB 227 and BG487 GAL4 lines)(n = 10, 8, 7, 8, 8, 6, 8, 6, 7, 9 and 7, respectively). ***p < 0.0001, one-way ANOVA with Tukey’s post comparison test, GAL4/Cat vs GAL4/+ . **(C)** Transient ubiquitous expression of catalase in tubPGAL80ts/UAS-Cat; daGAL4/+ larvae is sufficient to suppress UVC(M)-induced writhing behavior(n = 6 for all). ***p < 0.0001, one-way ANOVA with Tukey’s post comparison test, noHS vs HS. **(D)** Neuron-specific expression of antioxidant enzymes in mdIV neurons using ppk1GAL4 driver caused moderate suppression of writhing behavior in response to UVC(M). (n = 6, 4, 14, 5, 9, 4 and 6 respectively) **(E)** Writhing responses to UVC(H) could not be overcome by ubiquitous(daGAL4) or mdIV neuron-specific(ppk1GAL4) expression of antioxidants. **(F)** Overexpression of antioxidant enzymes had no effect on thermal nociceptive rolling response(n = 8, 6, 6 and 7, respectively). *p < 0.05, ***p < 0.001 from student t-test. For thermal nociception assays, data are presented as percentage of the total with each N value representing one individual larva tested. Data are presented as mean±SEM. For UVR-induced writhing behavior, each N value represents results from one experimental trial using 5 larvae exposed to UVR and visually scoring the percentage responding.

Both UVB and UVC have been associated with increased cellular ROS generation [21-23] and can induce strong larval writhing behavior (Figure 1A). To assess the possible role of ROS in UVR-induced behavior, we tested the ability of anti-oxidant enzyme overexpression to suppress the larval response. UAS-transposons expressing four different antioxidant enzymes, catalase (Cat), human catalase engineered to be secreted (hCat), human superoxide dismutase (hSod1) and methionine reductase A (MsrA), were tested by ubiquitous expression with daGAL4. Overexpression of Cat, hCat or MsrA resulted in a greatly reduced response to UVC(M) (Figure 2A), highlighting the importance of ROS generation. Interestingly, hSod1 failed to suppress the UVC(M) response even though it was shown to work well in the *Drosophila* system in previous studies [34,35]. Since Sod1 breaks down superoxide into the less toxic but longer-lived H_2_O_2,_ it is likely that H_2_O_2_ was actively produced in hSod1-overexpressing larvae upon UVR, and that this in turn led to robust writhing behavior. These results imply that H_2_O_2_ is a major mediator of mdIV neuron activation consistent with our previous studies showing that the mdIV neurons are activated by nanomolar levels of H_2_O_2_ in electrophysiological recording preparations [11].

UVC radiation is thought to penetrate poorly through human tissues [36]. In *Drosophila* larvae, UVC has been reported to act mainly in the cuticle [12]. However, our results demonstrate that the mdIV neurons, directly beneath the larval epidermal and cuticle layers mediate a behavioral response to UVC suggesting that UVC may affect deeper tissues. To identify larval tissues that contribute to ROS production in response to UVR, catalase was transgenically overexpressed using a collection of tissue-specific GAL4 lines (Figure 2B, Additional file 3: Figure S1). Overexpression of catalase in epidermal cells using two different drivers, NP6202 and GawB227, resulted in a greatly reduced response to UVC(M) with only 24% of larvae showing writhing behavior (Figure 2B).

Muscle cells are located in close proximity to mdIV neurons in the larval body wall and represent a significant mass that could either produce or absorb circulating ROS. Catalase overexpression using the muscle-specific BG487GAL4 transposon efficiently suppressed the UVC(M) response (Figure 2B). This result suggested that the larval muscle layers could potentially generate significant amounts of ROS although catalase overexpression in the dominant mass of the larval muscle layers could also function as a nonspecific sink capable of degrading large amounts of ROS. Overexpression of catalase in other cells using the GawBT98 and GawBc564 transposons had a much smaller effect on the induction of writhing behavior (Figure 2B), suggesting that epidermal and muscle cells are major sites for ROS generation in response to UVR.

Extensive recent work has shown that endogenous ROS play an essential role in numerous intracellular signaling pathways at low concentration [37,38]. Disruption of ROS signaling during earlier points of development could cause indirect phenotypic effects due to disruption of mdIV development. To investigate this possibility, endogenous levels of ROS were conditionally suppressed by overexpressing catalase just prior to UVC exposure. A tripartite system composed of GAL4, temperature-sensitive GAL80 (GAL80^ts^) and UAS-catalase was used to induce the temporal overexpression. GAL80^ts^ sequesters GAL4 and inhibits its transcriptional activity at low temperature but is inactivated when temperature is elevated to around 30°C, releasing GAL4 to promote UAS-dependent transcriptional activity [39]. Wandering third instar tubPGAL80^ts^/UAS-Cat; daGAL4/+ larvae were shifted to 30°C for 6 hours to allow ubiquitous catalase overexpression and then left at room temperature for 2 hours to acclimate before UVC(M) exposure. The larval writhing response was strongly suppressed by transient catalase overexpression with less than 25% of larvae exhibiting the writhing behavior (Figure 2C). This was essentially the same level of suppression shown in the continuous expression experiments (Figure 2A). The response of control animals not exposed to the temperature shift or lacking the UAS-Cat transposon was comparable to that in wild-type larvae (Figure 2C). This result suggests that continuous overexpression of antioxidant enzymes did not disrupt mdIV neuron development and emphasizes the acute nature of ROS-mediated induction of the writhing behavior.

Although tissue-specific expression experiments (Figure 2B) suggested that the epidermal layers of the larval body wall may be a prime source of ROS in response to UVR, we examined the effect of antioxidant enzyme overexpression in the mdIV neurons themselves using the mdIV neuron-specific *ppk1GAL4* transposon (Figure 2D). Overexpression of catalase, thioredoxin reductase (mitochondrial), and msrA in mdIV neurons caused a moderate suppression of the writhing response to UVC(M) (Figure 2D). Although this result suggests the possibility of an autonomous ROS response in the mdIV neurons themselves, it must be interpreted with caution since any manipulation, whether specific or nonspecific, that causes inactivation of the mdIV neurons would result in a suppression of the writhing response (Figure 1C). In addition, mdIV neuron-specific overexpression of antioxidants could potentially impact levels of H_2_O_2_ diffusing into the neurons from an external tissue source. mdIV neuron-specific antioxidant overexpression was unable to suppress the larval writhing response to UVC(H) (Figure 2E). Ubiquitous antioxidant expression also failed to suppress the response to UVC(H) (Figure 2E) suggesting that this high dose of UVC simply overwhelms the system for ROS degradation. mdIV neuronal morphology was examined in UAS-Cat/UAS-CD8GFP; ppk1GAL4/+ larvae to detect any potential developmental defects caused by mdIV neuron-specific expression of antioxidants (Additional file 4: Figure S2). No gross morphological defects were detected in the mdIV dendritic arbors suggesting that catalase overexpression does not cause nonspecific developmental defects in the mdIV neurons.

The mdIV neurons have been shown to play a crucial role in mediating thermal nociception behavior [9] in additon to UVR-induced nociception behavior (Figure 1C). The larval behavioral response to noxious heat (touched at midbody with a 42-45°C probe) was previously characterized as an intense rolling behavior and interpreted as an escape response [9,33]. *painless* mutants show reduced responses to both noxious heat [9,33] and UVR (Figure 1E) suggesting the possibility that these two sensory modalities may share certain molecular mechanisms. The possibility that ROS generation plays a roler in thermal nociception (Additional file 2) was assessed by observing the thermal nociception response in larvae ubiquitously overexpressing either Cat, hCat or MsrA (Figure 2F). Transgenic overexpression of these antioxidant enzymes strongly suppressed UVC(M)-induced writhing behavior (Figure 2A). Unlike the UVR-induced response, thermal nociception behavior was not affected by ubiquitous overexpression of antioxidant enzymes (Figure 2F), suggesting that ROS generation is not necessary for thermal nociception.

### UVR exposure increases levels of ROS in the larval bodywall

**Figure 3:**
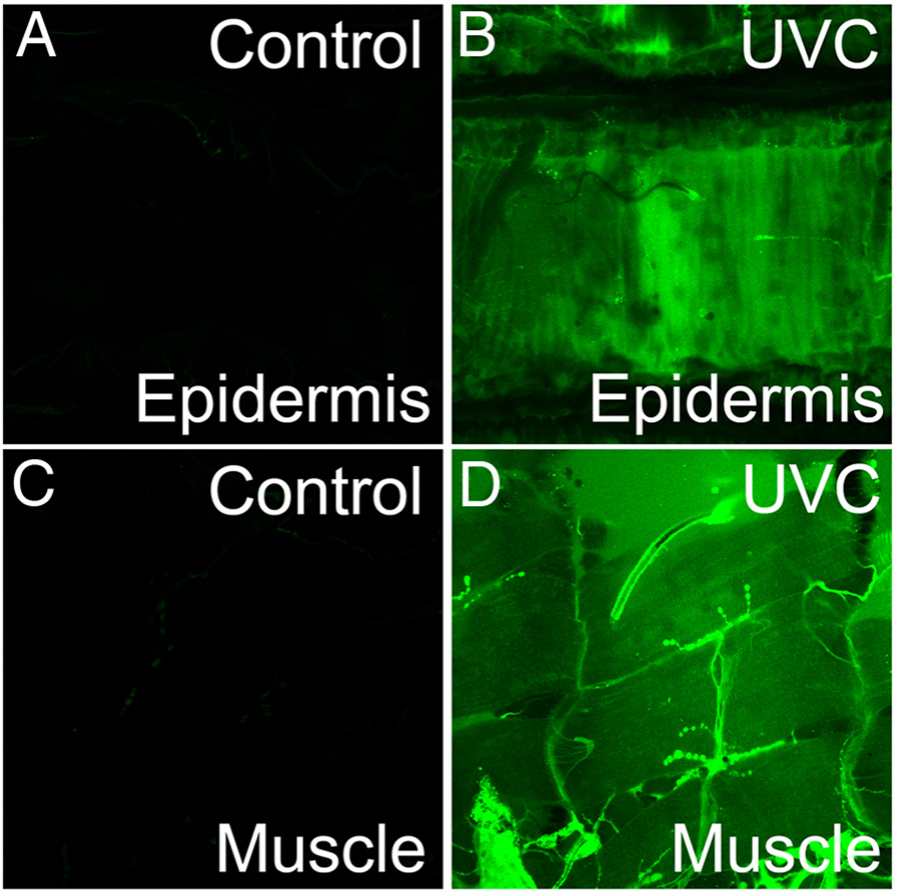
**Increased ROS levels in response to UVC irradiation.** Confocal microscope images of w^1118^ dissected larval body walls that were incubated with 10 μM carboxyl-H2DCFDA prior to UVC irradiation. **(AB)** Larval body wall images at epidermal focal plane. **(A)** Control wild-type (w[1118]) larvae showed no detectable fluorescence in the absence of UVC irradiation. **(B)** UVC-irradiated larval body walls displayed a significant increase in fluorescence in epidermal tissues indicating production of ROS in response to irradiation. **(CD)** Larval body wall images at muscle layer focal plane. **(C)** Wild-type controls showed no detectable fluorescence in the absence of UVC irradiation. **(D)** UVC-irradiated larval body walls displayed a significant increase in fluorescence in muscle layers indicating production of ROS in response to irradiation.

Predicted increases in tissue ROS levels in response to UVR were examined in larval body wall preparations incubated with 10 μM 2’,7’-dichlorodihydrofluorescein (carboxyl-H_2_DCFDA) prior to UVC exposure (40 mJ; 5 sec irradiation of 8 mW/cm^2^). Non-fluorescent H_2_DCF is converted into fluorescent 2′,7′-dichlorofluorescein (DCF) when exposed to an oxidative(ROS-containing) environment [40]. Confocal imaging of control larvae treated in an identical manner but not exposed to UVC showed no detectable fluorescence (Figure 3AC). After a brief exposure to UVC (40 mJ; 5 sec irradiation of 8 mW/cm^2^), larval preparations imaged at the focal plane of either the epidermis or muscle layer displayed a significant increase in fluorescence (Figure 3BD) consistent with increased levels of tissue ROS.

### UVR increases the activity of class IV md neurons

**Figure 4:**
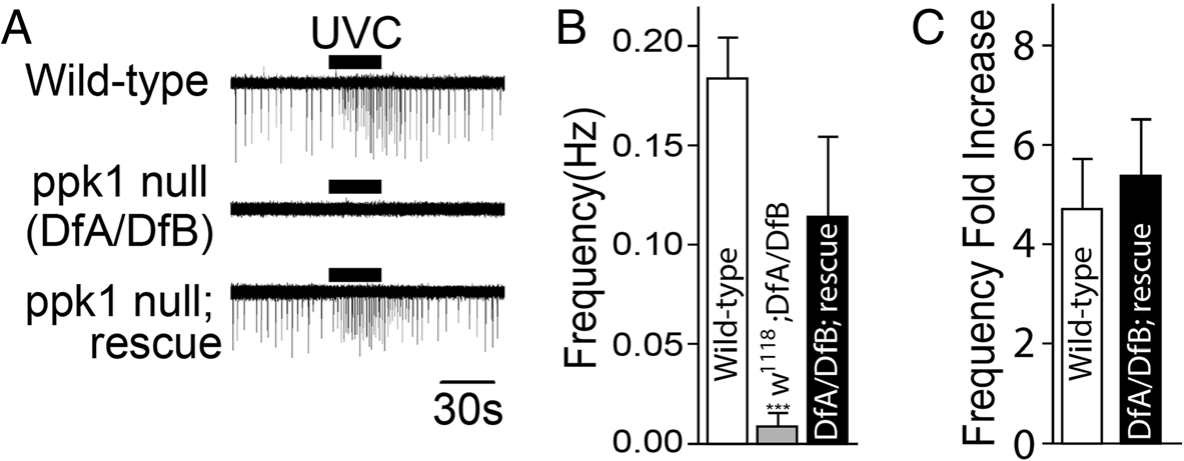
**Direct physiological activation of mdIV neurons by UVC. (A)** Representative single-unit extracellular recording traces from v’ada mdIV neurons in the larval body wall. Exposure to medium intensity UVC(M) increased the neuronal firing activity in wild-type neurons but *ppk1* null mutant (DfA/DfB) mdIV neurons failed to respond. The UVC(M)-induced response was rescued by transgenic expression of wild-type PPK1 in mdIV neurons. **(B)** Spontaneous basal spike frequency in wild-type and *ppk1* null mutant neurons. Spontaneous activity was restored by transgenic expression of wild-type PPK1 in mdIV neurons (n = 21, 13 and 8, respectively). ***p < 0.0001, one-way ANOVA with Tukey’s post comparison test vs wild-type. **(C)** Increase in mdIV neuronal firing rate in response to UVC(M) exposure represented a >4-fold increase from basal spontaneous firing frequency(n = 6 and 4, respectively). ***p < 0.001 from student t-test. All data are presented as mean±SEM.

Previous studies using a direct electrophysiological recording preparation demonstrated that mdIV neurons are activated by nanomolar levels of H_2_O_2_[11]. Using the same single-unit extracellular recording preparation, the electrophysiological response of the v’ada mdIV neuron in the lateral PNS cluster was tested for the direct activation of mdIV neurons by UVR (Figure 4A). Wild-type v’ada neurons exhibited spontaneous activity with a frequency of 0.2 Hz in the absence of stimulus (Figure 4A). Spontaneous activity in wild-type mdIV neurons was associated with use of low magnesium (4 mM) HL3 perfusion buffer. This low level of spontaneous activity was not seen when high magnesium (20 mM) HL3 was used in previous experiments characterizing mdIV neuron activation by H_2_O_2_[11]. The discharge rate of wild-type v’ada neurons was increased more than 4-fold in response to irradiation with UVC(M) (Figure 4).

Recordings from v’ada neurons in *ppk1* null (DfA/DfB) larvae revealed that they were silent with no detectable spontaneous activity (Figure 4A). Activation of *ppk1* null v’ada neurons by UVC(M) was strongly suppressed and this effect was rescued by transgenic expression of wild-type PPK1 (Figure 4AB). This result is consistent with those from behavioral experiments described earlier (Figure 2A).

### Noxious heat restores excitability of *ppk1* null mdIV neurons and induces discharges

**Figure 5:**
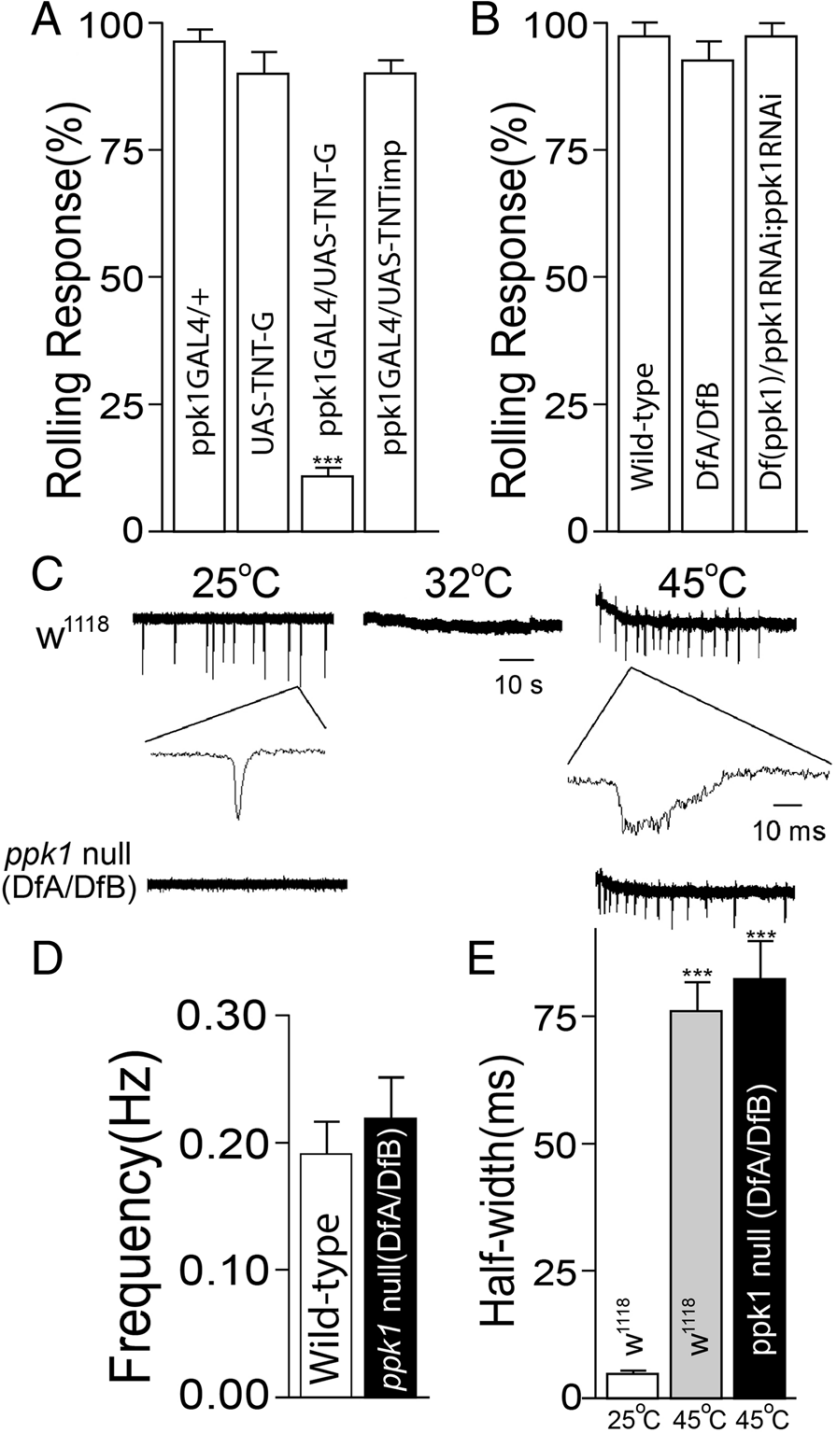
**Alteration in dynamics of mdIV neuron activation in response to noxious heat. (AB)** Larval noxious heat-induced rolling response. **(A)** mdIV neuron-specific expression of tetanus toxin (TNT-G) to block synaptic transmission strongly suppresses larval heat-induced behavioral response to noxious heat. (n = 7, 6, 16 and 10 respectively) ***p < 0.0001, one-way ANOVA with Tukey’s post comparison test vs ppk1GAL4/+ **(B)***ppk1* null mutant larvae (DfA/DfB) respond to noxious heat stimulus at levels comparable to wild-type (n = 16, 10 and 10, respectively). **(C)** Representative recordings of wild-type and *ppk1* null v’ada mdIV neurons at indicated bath temperatures. Noxious heat (45°C) activated both wild-type and *ppk1* null neurons with a spike frequency comparable to that in uninduced wild-type neurons (spontaneous activity) but with altered spike activation dynamics reflected as a broadened spike duration. **(D)** Comparable wild-type and *ppk1* null spike frequency at 45-46°C consistent with previous work demonstrating that PPK1 is not required for the mdIV neuronal response to noxious heat (n = 15 and 7, respectively). Not statistically significant by student’s t-test. **(E)** Comparison of wild-type and *ppk1* null mutant v’ada mdIV neuronal spike width at half maximum amplitude when recorded at 25°C vs 45°C. Wild-type and *ppk1* null neurons display comparable spike frequency and broadened spike duration when exposed to noxious heat. (n = 7, 6 and 7 respectively), ***p < 0.0001, one-way ANOVA with Tukey’s post comparison test vs w^1118^ (25°C). All data are presented as mean±SEM.

The absence of spontaneous activity in *ppk1* null (DfA/DfB) mdIV neurons and the transgenic rescue of spontaneous activity suggest an overall reduction in excitability in the absence of PPK1 (Figure 4A). However, *ppk1* null mutant larvae have been shown to respond normally to noxious heat stimulus [10] (also see Figure 5B) which would not be consistent with a general role. The ability of noxious heat to induce mdIV activity in the absence of PPK1 was examined using the v’ada mdIV single-unit extracellular recording preparation [11]. A noxious heat stimulus was applied by raising the bath temperature to 45-46°C. Wild-type v’ada mdIV neurons exhibited spontaneous activity at room temperature as described earlier (Figures 4A, 5C) but showed a complete suppression of spontaneous activity when bath temperature reached 32-33°C (Figure 5C). Bath temperatures of 45-46°C, corresponding to a noxious heat stimulus, elicited increased v’ada mdIV neuron firing activity (Figure 5C) consistent with previous studies demonstrating a role for the v’ada mdIV neurons in thermal nociception [9,33].

In contrast to the increase in mdIV neuron firing rate demonstrated for UVR-mediated activation (Figure 4AC), the rate of discharge elicited by noxious heat at 45-46°C did not differ significantly from the rate of spontaneous firing at room temperature (Figures 4AB and 5CD). This result suggested the possibility that noxious heat-induced mdIV neuron activation, the basal spontaneous activity and UVR-induced activation may each be encoded differently. Previous work in other sensory systems, such as olfaction, has demonstrated that firing rate and/or amplitude are not the only parameters used for determination of stimulus coding. Results suggest that the broad variety of odor stimuli are encoded by transient dynamics and odor-specific latencies independently of stimulus intensity [41,42].

Detailed analysis of neuronal spikes elicited by noxious heat showed that spike duration at 45°C was much broader than that of basal spontaneous activity (Figure 5C). Comparison of spike width at half maximum amplitude revealed a striking difference between heat-induced impulses (~75 ms) and spontaneous spiking (~5 ms) (Figure 5CE). Although a more detailed analysis will be necessary, these results together with the increase in discharge rate induced by UVR, suggest that the mdIV neurons may encode the neuronal responses in multiple ways depending stimulus type.

Previous studies have demonstrated that mdIV neuronal PPK1 is not necessary for the noxious heat-induced behavioral response [10]. The single-unit extracellular recording preparation was used to assess a potential role for PPK1 in the noxious heat-induced neuronal response. Although *ppk1* null v’ada mdIV neurons did not exhibit spontaneous activity at room temperature (Figure 5C), they displayed a strong response to noxious heat, with an impulse frequency of, ~0.2 Hz, which is comparable to that of wild-type (Figure 5CD). Spike width at half maximum amplitude (~75 ms) was also comparable to that of wild type (Figure 5E). This finding is consistent with results from our behavioral assays (Figure 5B) and in published studies [10] demonstrating that PPK1 was dispensable for noxious heat-induced rolling behavior.

## Discussion

The mdIV sensory neurons, innervating the larval body wall with a complex dendritic arbor, play a key role in cellular and molecular mechanisms mediating the larval behavioral response to UV irradiation. Results presented here indicate that the mdIV neurons are responsible for detecting UVR and initiating the larval writhing motion response. Consistent with previous studies demonstrating an acute sensitivity of mdIV neurons to nanomolar levels of H_2_O_2_[11], our results demonstrate a role for endogenous ROS in mediating the UVR response.

### Significance of UV nociception behavior

Noxious thermal and mechanical stimuli elicit immediate behavioral responses from essentially all multicellular organisms [43,44]. These behavioral responses play a role in preventing tissue damage by preventing prolonged exposure to noxious stimuli. Unlike thermal and mechanical stimuli, UVR does not appear to elicit such an immediate and protective behavioral response from mammals even though it can cause a devastating effect at high doses. However, certain insects and nematodes display an immediate behavioral response to UVR. For example, the American cockroach quickly escapes the site of UVR exposure by moving to a shaded area [45]. Even *C. elegans* accelerates locomotion away from UV light even though they have no functioning visual system. This behavior has been proposed to be a protective mechanism against prolonged exposure to UV that can paralyze and kill the organism [46]. Results presented in this and other studies [13] suggest that *Drosophila* larvae are also programmed to respond immediately to UVR in a visual system-independent manner.

It is not clear why the immediate aversive behavioral response to prolonged UV exposure is found only in lower animals though we hypothesize that it relates to the fact that the integument system of insects and nematodes is much simpler in structure compared to that of vertebrates. The former is composed of a single layer of epidermal cells covered with cuticle and the latter consists of multiple layers of heterogeneous cell types. The simpler integument system may allow UVR to penetrate deeper into the body UV into the body with potential for more detrimental effects to the whole organism. In support of this idea, a low dose of UV (slightly above 20 mJ/cm^2^) is sufficient to kill *Drosophila* larvae [30]. This is far below the dose of 250–1000 mJ/cm^2^ which merely sensitizes the sensory neurons in rats [47]. This finding is consistent with an increased susceptibility of insects and other small animals with simple integument system to the devastating effects of UVR.

It is useful to compare the experimental UVR exposure applied in our studies with what might be considered a normal UVR exposure from natural sunlight. As discussed earlier, the primary UVR allowed to reach the earth’s surface are UVA and UVB. UVA passes through the atmosphere with little diminution and ~90% of UVB is blocked by atmospheric ozone absorption [18-20]. UVC is totally blocked by an intact ozone layer surrounding the earth. An accurate UVR dosage can be difficult to determine since it depends upon latitude, time of day, atmospheric conditions and UVR wavelength. In recent years, attempts have been made to standardize UVR exposure as a measure of UVR levels necessary to elicit skin inflammation or erythema [48,49]. This is referred to as a standard erythena dose (SED). One SED has been designated as UVR equivalent to an exposure of 100 Jm^-2^. UVR exposure is routinely referred to as SED/hr with a mean dose ranging from 5–7 SED/hr during daylight hours depending upon latitude. The normal exposure on a clear summer day in Europe is ~30-40 SED [48]. Converting our dose of UVC(M) (1.2m Wcm^-2^) to Jm^-2^ using a standard conversion (1 mWcm^-2^ = 10 J/sm^2^ with a 5 second exposure) yields 60 Jm^-2^ or 0.6 SED. This is then equivalent to outdoor exposure of ~6 min in full sunlight. However, experimental conditions using UVC were designed as a mechanism to produce endogenous ROS in larvae and not to mimic natural sunlight since little UVC actually reaches the earth’s surface as part of natural sunlight.

### ROS-mediated neuronal activation

Both constant and acute expression of antioxidant enzymes greatly suppressed UVR-induced writhing behavior (Figure 3), highlighting the importance of ROS generation in this response. This is consistent with previous studies demonstrating that mdIV neurons are activated by nanomolar levels of H_2_O_2_[11]. The response to H_2_O_2_ exposure was immediate and application of H_2_O_2_ to a local dendritic field was sufficient for mdIV neuronal activation suggesting that H_2_O_2_ is a direct activator of the class IV md neurons [11].

Accumulating evidence suggests that ROS can induce neurons to either fire or increase their rate of firing in the absence of other stimuli. H_2_O_2_ has been shown to stimulate the capsaicin-sensitive vagal lung afferents of rats [50,51]. The same study demonstrated that transient receptor potential vanilloid 1(TRPV1) receptors and P2X purinoreceptors were responsible for the activation of these afferents by H_2_O_2_. Capsaicin-sensitive cardiac vagal and sympathetic afferents are also known to be activated by H_2_O_2_ in rats [52]. Since the capsaicin sensitivity is a hallmark of nociceptive sensory neurons [53], these results implicate ROS as an efficient activator of nociceptors. In a study examining the ROS sensitivity of afferent neurons in rat Splanchnic fibers, a total of 52 units were identified and each was first examined for its sensitivity to bradykinin, mechanical and thermal stimuli before being tested for responsiveness to H_2_O_2_[54]. Units sensitive to both mechanical and thermal stimuli showed the strongest response to H_2_O_2_. This is similar to results from larval mdIV neurons that are responsive to multiple forms of noxious stimuli including thermal, mechanical and ROS. However, some afferents of the Splanchnic C fibers were only responsive to H_2_O_2_. Based on the observation that these fibers contain neuropeptides known to influence the respiratory burst, the H_2_O_2_-specific neurons have been proposed to play a role in detecting ROS generated during inflammation.

### Role of PPK1

The mdIV sensory neurons have been implicated in multiple biological phenomena including thermal and mechanical nociception, light-avoidance and behavioral transitions occurring at the late 3rd instar stage [7-10,55]. Loss of PPK1 expression has led to defects in mechanical nociception [10], area-restricted searching (ARS) [7], intermediate surfacing transition (IST) [55] and thermal preference behaviors [10]. In addition, results presented here have revealed an essential role for PPK1 in the response to UVC of medium intensity. PPK1 has, however, been shown to be dispensable for thermal nociceptive behavior [9] and writhing motion induced by high intensity of UVC (this study). Collectively, these results indicate that PPK1 contributes to the ability of mdIV neurons to function in a complex context-dependent manner.

It remains unclear how *ppk1* null mdIV neurons selectively affect certain biological processes. Answering this question represents a challenge that is inherent to the characterization of most polymodal nociceptors. Molecular and physiological studies are often performed with the goal of characterizing the role of a single molecule or protein in polymodal nociceptor function. However, nociceptor neurons express numerous ion channels, transmembrane receptors and a variety of signaling molecules all of which must be coordinated to produce a uniform neuronal output. Although our understanding of the molecular components of somatosensory signaling has made great leaps over the past decade, we still lack a full understanding of how all of these molecules interact with each other in the context of a polymodal nociceptor. Although PPK1 has been implicated in a number of sensory processes, suggesting that it may serve as a receptor for multiple types of sensory stimuli, this may be unlikely considering the fact that sensory receptive molecules are usually specific for a single stimulus type. Alternatively, PPK1 may act to regulate the general excitability of neurons to modify neuronal sensitivity to a variety of stimuli. In our experiments, *ppk1* null mutant mdIV neurons displayed reduced excitability as reflected in the absence of spontaneous activity.

It should be emphasized that our results do not allow any conclusions to be reached as to whether or not the PPK1 protein is itself being modified by increased ROS levels. Although the large extracellular domain of the PPK1 DEG/ENaC subunit contains several cysteine-rich regions that could potentially be subject to ROS-mediated modification [6], that has not yet been tested. Numerous reports have described the role of O_2_ and ROS in modulation of vertebrate ENaC and ASIC activities [56-63]. Therefore, the endogenous target(s) of ROS-mediated signaling in mdIV neurons may be PPK1 itself or another heterologous protein functioning in concert with rather than directly on PPK1.

This raises the question of whether the presumed increase in ROS levels in response to UVR “activates” the mdIV neurons or instead “sensitizes” them. Our previous work suggests that the sensitivity of mdIV neurons to H_2_O_2_ is developmentally regulated with a >100-fold loss of sensitivity between 78 and 96 h after egg laying (AEL) [11]. This time period corresponds to the developmental shift during the third larval instar when *D. melanogaster* larvae exit the food source prior to pupariation. Despite this significant loss of H_2_O_2_ sensitivity, late third instar larvae require PPK1 function during selection of a pupation site to avoid dry surfaces that lead to frictional stranding and death by dessication [64]. These results are consistent with an increase in ROS levels following exposure to either UVR or atmospheric oxygen serving to hypersensitize the mdIV neurons and their PPK1-mediated mechanosensory response contributing to sustained larval food immersion during foraging stages. Recent results examining homeostatic plasticity at the larval neuromuscular junction (NMJ) have identified two other *Drosophila* DEG/ENaC family members, pickpocket11 (ppk11) and pickpocket16 (ppk16) required in presynaptic motoneurons as modulators of presynaptic neurotransmitter release at the larval NMJ [65]. These DEG/ENaC subunits appear to control presynaptic membrane voltage to control calcium channel activity and neurotransmitter release. These results suggest that the functional relationship between different ion channel structural families is complex and that the diverse family of DEG/ENaC/ASIC channels is also capable of diverse roles in regulating neuronal activity. These key questions concerning mechanonociceptor sensitivity and function are universal challenges in common with vertebrate models. The mdIV sensory neurons should serve as an excellent genetic model to better understand these processes.

## Conclusions

Results presented here demonstrate that the writhing response to UVC irradiation observed in *Drosophila* larvae is mediated by generation of ROS species capable of activating mdIV mechanonociceptors in the larval body wall. This establishes the role of ROS species as endogenous signaling molecules in the larval body wall and as modulators of neuronal activity controlling stereotypical behavioral responses to changes in the external environment.

## Methods

### Fly strains

Flies were raised at 25°C on standard cornmeal-yeast-agar medium. The w^1118^ stock was used as a wild-type control in all experiments. The overlapping deficiency stock, w; Df(2 L)b88h49/Df(2 L)A400, was used as a *ppk1* null stock as previously described [8] and is designated as DfA/DfB. The *ppk1GAL4* transposon was used to drive expression in mdIV neurons (Ainsley et al., [8]). Other fly strains used include: GMRGAL4, GMR-hid, tubGAL80^ts^, 227GAL4, T98GAL4, c564GAL4, UAS-TNTg, UAS-TNTimp (Blooming Drosophila Stock Center); NP6202 (National Institute of Genetics, Japan); BG487GAL4 [66]; UAS-catalase and UAS-hsod1 [67]; UAS-hcat [68]; UAS-msrA (Toshi Hoshi); pain^1^, pain^2^, pain^3^ and pain^GAL4^[33].

### UV-induced writhing behavior

Larvae were incubated at room temperature for 2 hours before all behavioral assays. For the assay of UV-induced writhing behavior, five early wandering stage larvae (96 h AEL) were taken from a vial, briefly rinsed in dH_2_O and placed in a petri dish. Larvae were allowed to acclimate to the new environment for 5 minutes and then exposed to a UV light source with the appropriate band pass filters to generate UVA (360 nm), UVB (312 nm) and UVC (254 nm). For tests of varied UVC intensity, a UV light source with the specified intensity (according to manufacturer specifications) was used with a UVC (254 nm) band pass filter. A DNA document system from UVP (8 mW/cm^2^ according to manufacture’s manual) was used for high intensity UVC(H). A hand-held UV lamp was used for medium intensity UVC(M) exposure (1.2 mW/cm^2^ when irradiated from 7 cm) and a portable UV lamp from Fisher Scientific (0.17 mW/cm^2^) was used for low intensity UVC(L) exposure. Larvae displaying writhing behavior within 5 seconds of UV exposure were scored as a positive writhing response. Each N value represents one group of 5 larvae. Results from two consecutive experiments were combined to represent one trial and the data are expressed as the percentage responding. In experiments involving the temporal expression of catalase, the larvae carrying tubGAL80^ts^, daGAL4 and UAS-cat were heat-shocked at 30°C for 6 hrs and allowed to recover from heat-shock at RT for 2 hr before the UVR assay was carried out.

### Thermal nociception behavior

For each trial, eight wandering stage larvae were removed from a vial and briefly rinsed in dH_2_O. After rinsing, larvae were placed on a water-smeared agar-plate and allowed to acclimate to the new environment. After 10 minutes, one larva at a time was touched on the dorsal side with the blunted tip of a tweezer bent to 90° and pre-heated to 42-43°C. Larvae that initiated rolling motion within 20 seconds were scored as responsive. Data are presented as percentage of the total with each N value representing one individual larva tested.

### Imaging of larval ROS levels

Larval body walls were dissected for irradiation and imaging as larval filets with internal organs and brain removed. Larval body walls were incubated with 10 μM carboxyl-H2DCFDA in PBS with agitation for 5 min. Tissues were irradiated immediately with 40 mJ UVC (corresponding to 5 sec irradiation of 8 mV/cm^2^) using a Stratagene UVC DNA-linker. After irradiation, body walls were further incubated with 10 μM carboxyl-H2DCFDA in PBS with agitation for 10 min at RT. Images were acquired using an LSM710 confocal microscope with a FITC filter set. Control larval body wall preparations were prepared and treated identically except for the absence of UVC irradiation.

### Dissection and electrophysiological recording

Extracellular electrophysiological recordings were carried out as previously described [11]. Dissected larval body wall preparations were perfused with low magnesium HL3 solution containing (in mM) 70 NaCl, 5 KCl, 1.5 CaCl_2_, 4 MgCl_2_, 10 NaHCO_3_, 5 Trehalose, 115 Sucrose and 5 HEPES. The low magnesium HL3 is known to better support neuronal activity [69].

## Competing interests

The authors declare that they have no competing interests.

## Authors’ contributions

M-JK conceived the study, carried out all of the experiments and participated in the drafting and editing of the manuscript. WAJ participated in the conception and design of the experiments, analysis of and interpretation of data, as well as the drafting and editing of the manuscript. Both authors read and approved the final manuscript.

## Supplementary Material

Additional file 1 **Larval writhing behavior in response to UVC(H) irradiation.** Video showing an individual ppk1GAL4/+ control larva on an agarose plate exposed to UVC(H) irradiation at the indicated timepoint. Larva shows strong writhing behavior instaneous to UVC(H) exposure onset. Subsequent frames show an individual ppk1GAL4/UAS-TNT-G larva exposed to the same dose of UVC(H) irradiation at the indicated timepoint. Transgenic inactivation of mdIV neurons in the ppk1GAL4/UAS-TNT-G larva causes a complete suppression of the writhing response. Click here for file

Additional file 2 **Larval writhing behavior in response to noxious heat.** Video showing an individual ppk1GAL4/+ control larva touched at midbody length with a probe heated to 42°C. The wild-type control larva shows a strong writhing behavior response as previously reported [33]. Subsequent frames show an individual ppk1GAL4/UAS-TNT-G larva exposed to the same noxious heat probe at the indicated timepoint. Transgenic inactivation of mdIV neurons in the ppk1GAL4/UAS-TNT-G larva causes a complete suppression of the writhing response. Comparison of noxious heat induced writhing with UVC(H) induced writhing (Additional file 1) shows that the two responses are very similar if not identical. Click here for file

Additional file 3: Figure S1 Expression patterns of tissue-specific GAL4 driver transposons. All stocks carrying tissue-specific GAL4 driver transposons were crossed with UAS-mCD8GFP to visualize and evaluate expression patterns. Indicated tissues were dissected from GAL4/UAS-CD8GFP larvae and imaged using an LSM710 confocal microscope. Click here for file

Additional file 4: Figure S2 Effects of catalase overexpression on mdIV dendritic morphology. mdIV dendritic morphology in (A)ppk1GAL4/UAS-mCD8GFP or (B) ppk1GAL4/UAS-mCD8GFP; UAS-Cat/+ larvae. Confocal images of GFP fluorescence were obtained from living larvae to represent the overall dendritic arbor of two adjacent mdIV neurons. No gross defects in dendritic morphology are detected after catalase overexpression in mdIV neurons. Click here for file
