# Supplementary figures and images for: ROS-mediated activation of Drosophila larval nociceptor neurons by UVC irradiation
Source: BMC Neurosci. 2014 Jan 16;15:14. doi: 10.1186/1471-2202-15-14 (PMC3898224; doi:10.1186/1471-2202-15-14)

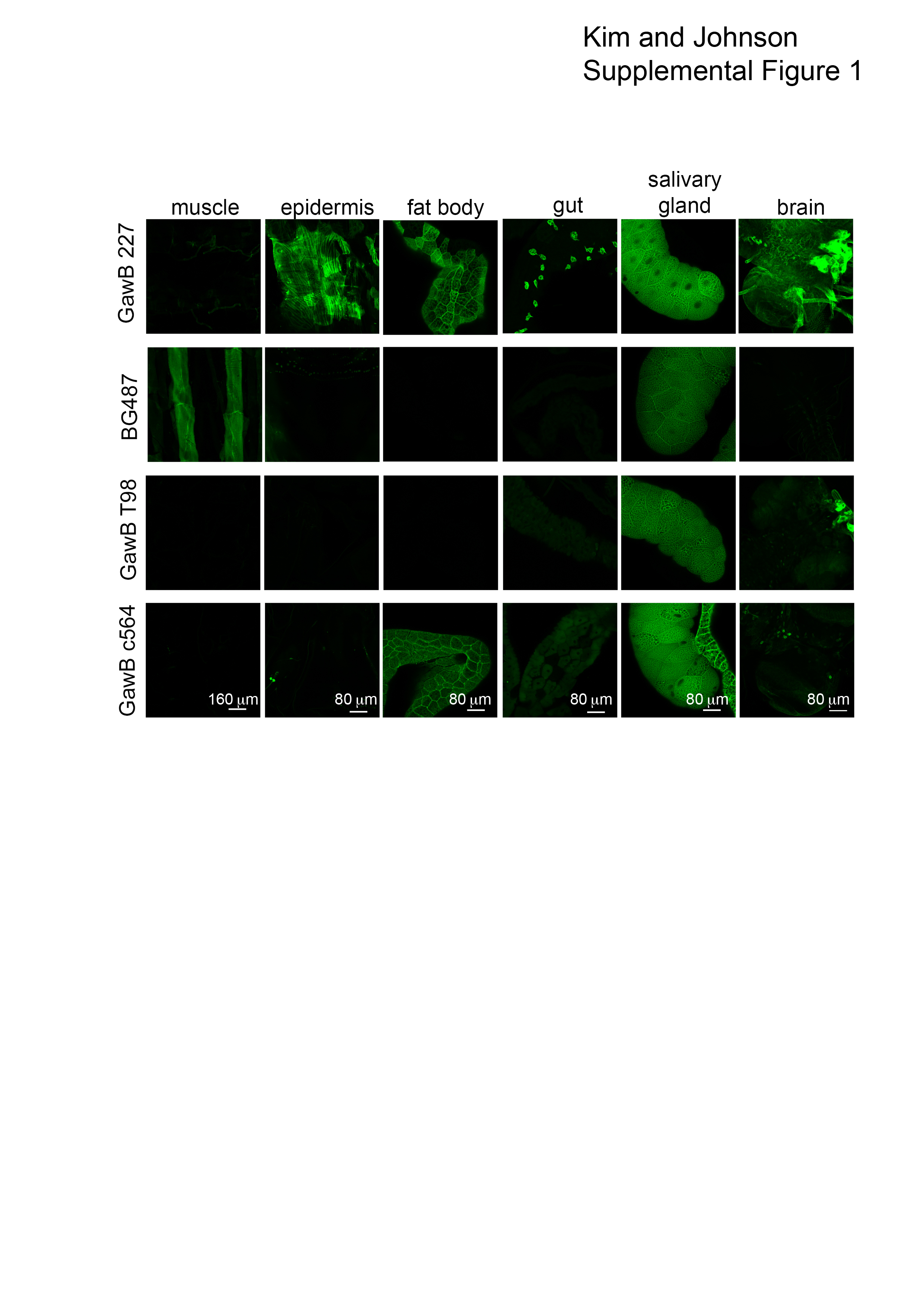

Supplement: Additional file 3: Figure S1 — Expression patterns of tissue-specific GAL4 driver transposons. All stocks carrying tissue-specific GAL4 driver transposons were crossed with UAS-mCD8GFP to visualize and evaluate expression patterns. Indicated tissues were dissected from GAL4/UAS-CD8GFP larvae and imaged using an LSM710 confocal microscope. [file 1471-2202-15-14-S3.jpeg]

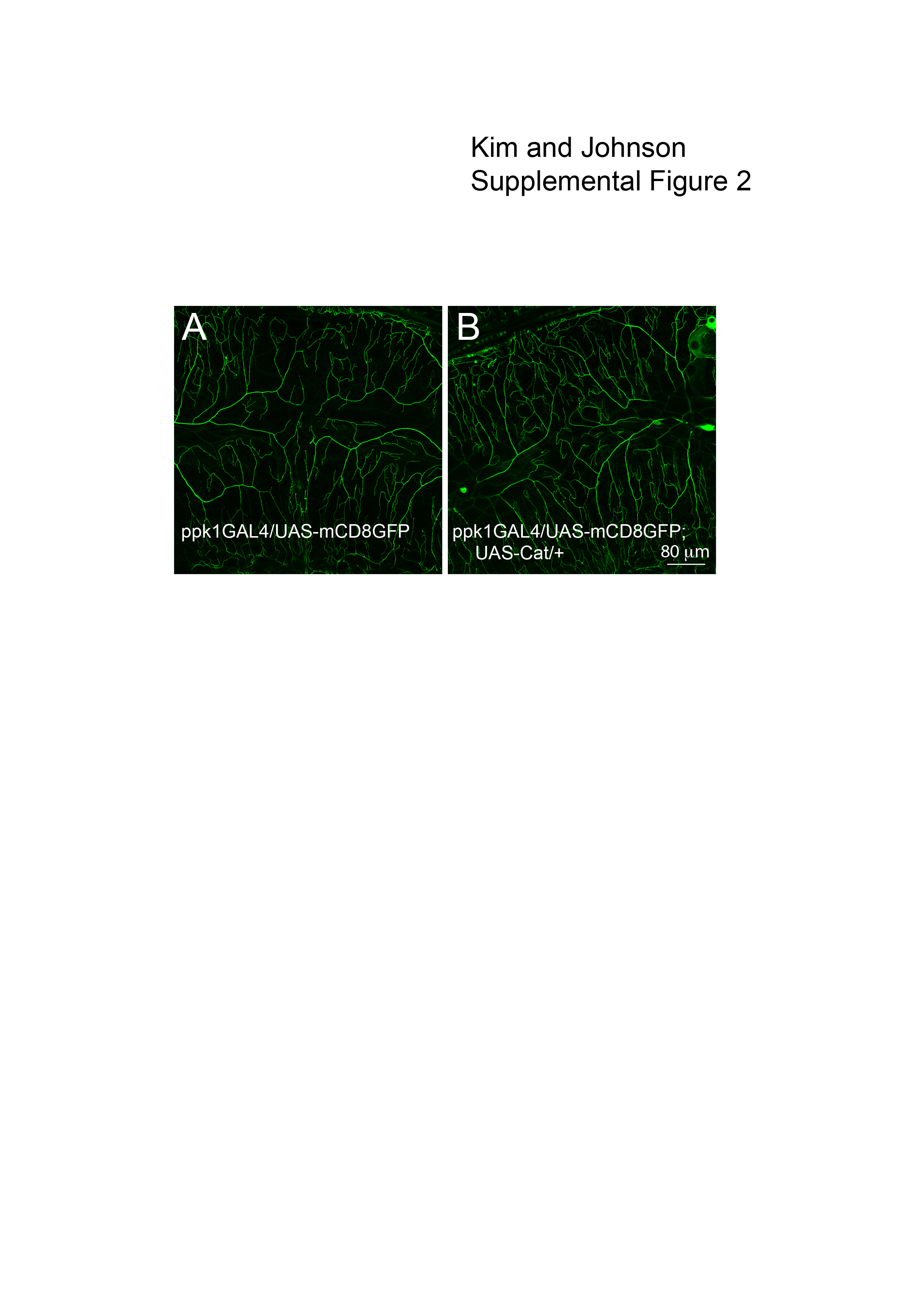

Supplement: Additional file 4: Figure S2 — Effects of catalase overexpression on mdIV dendritic morphology. mdIV dendritic morphology in (A)ppk1GAL4/UAS-mCD8GFP or (B) ppk1GAL4/UAS-mCD8GFP; UAS-Cat/+ larvae. Confocal images of GFP fluorescence were obtained from living larvae to represent the overall dendritic arbor of two adjacent mdIV neurons. No gross defects in dendritic morphology are detected after catalase overexpression in mdIV neurons. [file 1471-2202-15-14-S4.jpeg]
